# APOSCREEN-1 – a prospective, single-arm clinical trial for the implementation of a pharmacy-based screening for cardiovascular-kidney-metabolic risk factors in Schleswig-Holstein

**DOI:** 10.1186/s12882-026-05090-x

**Published:** 2026-06-05

**Authors:** Eric Amelunxen, Amelie Kokot, Benedikt Kolbrink, Sarah-Yasmin Thomsen, Maja L. Meßtorff, Matthias Laudes, Kenny R. Lienhard, Lucas M. Bachmann, Laura K. Sievers, Roland Schmitt, Kevin Schulte, Friedrich A. von Samson-Himmelstjerna

**Affiliations:** 1 Department of Internal Medicine IV Nephrology and Hypertension, University Hospital Schleswig-Holstein, Campus Kiel, Kiel, Germany; 2 Department of Internal Medicine I, Division of Endocrinology, Diabetes and Clinical Nutrition, University Hospital Schleswig-Holstein, Campus Kiel, Kiel, Germany; 3 Institute of Diabetes and Clinical Metabolic Research, University Hospital Schleswig-Holstein, Campus Kiel, Kiel, Germany; 4 Medignition AG, Research Consultants, Zurich, Switzerland; 5 Epidemiology, Biostatistics & Prevention Institute (EBPI), University of Zurich, Zurich, Switzerland

**Keywords:** Prevention, Cardiovascular risk, Chronic kidney disease, Diabetes, Hypertension, Point-of-care testing, Community pharmacy, Public health

## Abstract

**Background:**

Cardiovascular-kidney-metabolic (CKM) syndrome has emerged as a major global health burden and driver of cardiovascular disease (CVD), the leading cause of death worldwide. Effective management of CKM depends on timely identification of underlying risk factors. Nevertheless, participation rates in primary care-based screenings are low. Consequently, CKM syndrome oftentimes remains undetected until organ damage is clinically present. Community pharmacies offer an accessible, yet underused, setting to enhance early detection. APOSCREEN-1 evaluates the feasibility and diagnostic yield of a pharmacy-based multi-parametric screening for cardiovascular-kidney-metabolic health.

**Methods:**

APOSCREEN-1 is a prospective single-arm clinical trial conducted in 20 community pharmacies in the German state of Schleswig-Holstein. Adults (*n* = 1000) aged ≥ 40 years with predefined risk criteria are included. Participants undergo standardized point-of-care testing (glycated hemoglobin, lipid profile, urinary albumin, blood pressure). Additionally, clinical history is assessed and results are transmitted to the study center via an online platform. Patients meeting pre-defined thresholds of the tested parameters are followed up by confirmatory laboratory testing at the study center or at the participants’ general practitioner. Primary outcomes include completion rate, implementation metrics from the pharmacy perspective, and the number needed to screen to detect unknown or insufficiently managed cardiometabolic risk factors. Secondary outcomes comprise participant metrics, diagnostic metrics of the screening, evaluation of the clinical impact.

**Discussion:**

This study aims to address the unmet need for scalable prevention of CVD by identification of CKM syndrome risk factors outside traditional primary care settings. Evidence on feasibility, acceptance, and diagnostic benefit may support the use of community pharmacies as an additional access point for early CKM syndrome detection. Future interventional studies will be required to evaluate structured follow-up pathways and long-term effectiveness.

**Trial registration:**

This study was registered with the German trial registry (Deutsches Register klinischer Studien) on 29.01.2026, under trial number DRKS00039149.

**Supplementary Information:**

The online version contains supplementary material available at 10.1186/s12882-026-05090-x.

## Background

Cardiovascular disease (CVD) remains the leading cause of death worldwide [1]. Its development is driven predominantly by cardiovascular risk factors, including obesity, smoking, arterial hypertension, diabetes mellitus, and hypercholesterolemia. Chronic kidney disease (CKD) further amplifies cardiovascular risk and represents an independent risk factor. The interplay and combination of these factors have been defined as cardiovascular-kidney-metabolic (CKM) syndrome [2, 3].

The prevalence of the CKM syndrome is rising substantially [4]. In industrialized countries, 6–13% of the population are affected by advanced CKM syndrome stages involving organ damage, which corresponds to an estimated 5–11 million individuals in Germany. In the long-term, CKM syndrome shortens life expectancy, reduces social or occupational participation and is a major public health burden [5, 6].

Germany exemplifies this trend: despite the highest healthcare expenditure in the EU (11,7% of GDP in 2023), life expectancy remains below average due to CVD [7, 8]. Currently ranked only 17th out of 27 EU countries, Germany’s average life expectancy of 81.1 years has fallen below the EU average since 2010. This discrepancy suggests that Germany’s healthcare system fails to convert spending into better health outcomes.

Early identification of CKM syndrome is essential to avoid organ damage [9, 10]. In Germany, a nationwide preventive health check-up program exists to facilitate early detection of CKM syndrome and its contributing conditions. However, despite the long-standing availability of such programs, participation rates remain low with only 50% of the German population participating regularly [11]. Therefore, a large proportion of individuals remain undiagnosed until clinical manifestation occurs: approximately 49–84% of CKD cases, 20% of arterial hypertension, 22% of diabetes mellitus, and more than 50% of dyslipidemia cases remain undetected in the general population [12–14]. This underscores the urgent need for more effective and accessible screening strategies.

The approximately 16.800 German community pharmacies represent a trusted healthcare resource for the public, and are frequently visited with 64% of the population visiting at least once per month [15]. Pharmacists are highly trained healthcare professionals that may support the healthcare system with clinical services but remain an underutilized resource in this regard.

The APOSCREEN-1 concept, developed in collaboration with the Pharmacists’ Association of Schleswig-Holstein, aims to address this critical gap in secondary prevention by implementing a low-threshold, risk-based, and multi-parametric screening approach in community pharmacies.

## Methods/design

### Study aim and design

The APOSCREEN-1 study is a single-arm clinical trial conducted in community pharmacies located in the federal state of Schleswig-Holstein, Germany. The study aims to assess the feasibility and diagnostic yield of a multi-parametric, pharmacy-based, risk-adapted point-of-care (PoC) screening for CKM syndrome risk factors. It follows a two-step approach: first, a pragmatic point-of-care screening in pharmacies is conducted to triage patients with high cardiovascular risk who may benefit strongly from further diagnostic work-up. In a second step, these patients undergo detailed confirmatory testing and receive guideline-based therapeutic recommendations. Ethical approval has been obtained from the local institutional review board (AZ D616/25).

### Study setting and pharmacy selection

The study takes place in the outpatient setting with a planned number of 20 participating pharmacies. This number was chosen pragmatically for the pilot implementation phase to balance sufficient recruitment capacity with feasibility of onboarding, monitoring, technical support, and confirmatory follow-up. Each pharmacy is expected to recruit approximately 50 participants, resulting in a total planned sample size of 1,000 participants.

Pharmacies will be selected in collaboration with the Pharmacists’ Association of Schleswig-Holstein. The Association invited community pharmacies in Schleswig-Holstein to express interest in participation. From this pool of interested pharmacies, sites will be selected purposively according to predefined implementation-relevant criteria. First, we aim to include 10 pharmacies located in the region of the study center and 10 pharmacies located at greater distance from the study center. Second, within each of these two groups, we aim to include five pharmacies with customer restroom facilities allowing on-site urine albumin-creatinine ratio (UACR) testing and five pharmacies without such facilities, resulting in 10 pharmacies with and 10 pharmacies without on-site UACR testing capacity. Third, pharmacies will be selected to ensure broad geographic coverage across Schleswig-Holstein, including northern, southern, western, and eastern regions of the federal state.

This selection strategy is not based on random sampling and is not intended to generate a representative sample of all German community pharmacies. Rather, it is a purposive implementation-oriented sampling strategy designed to capture relevant heterogeneity in pharmacy infrastructure, distance to the study center, and regional accessibility within Schleswig-Holstein.

### Primary outcomes

The study will assess three primary outcomes:

(I) Participant-level feasibility is assessed by screening completion rates of participants who provided informed consent. The outcome is defined as completion of the structured questionnaire, performance of blood pressure measurement and PoC-testing for dyslipidemia and HbA1c. The study aims for an overall completion rate ≥ 70% based on the reported use of primary care screenings and prospective feasibility on the national level in Germany.
(II) Implementation outcomes from the pharmacy perspective are evaluated for the implementation domains acceptability, feasibility, and sustainability. The outcome is evaluated using validated instruments, including the Acceptability of Intervention Measure (AIM), the Feasibility of Intervention Measure (FIM), and the Normalization Measure Development Questionnaire (NoMad) to assess sustainability (Supplementary Tables 1 and NoMad Questionnaire) [16–19]. These instruments use standardized self-administered questionnaires completed by pharmacy staff that are rated on Likert scales. Validated German versions will be used. For each domain, the outcome criterion is defined by a null hypothesis assuming a median Likert-scale score ≤ 3. Rejection of the null hypothesis will be interpreted as evidence of sufficient implementation performance under routine practice conditions.
(III) The medical screening yield is quantified by the number needed to screen (NNS) to identify at least one previously unknown or insufficiently managed CKM syndrome risk factor. These are defined as non-HDL cholesterol ≥ 5.7 mmol/L for known dyslipidemia or lipid-lowering therapy, HbA1c ≥ 8.5% in participants with known diabetes mellitus, positive albuminuria test without RASi or SGLT2i therapy in participants with CKD, or blood pressure ≥ 140/80 mmHg for participants with known hypertension. A NNS ≤ 5 will be considered as indicative of a clinically meaningful detection efficiency of the multi-parametric screening approach. Further, a NNS between 5 and 10 will be considered an exploratory finding indicating the need for potential refinement or modification of the screening strategy. An observed NNS > 10 will be interpreted as an unfavorable feasibility outcome under routine practice conditions.

An NNS of ≤ 5 was predefined as a pragmatic threshold for clinically meaningful detection efficiency. This threshold reflects the identification of one previously unknown or insufficiently managed actionable risk factor in 20% of screened at-risk individuals. Although this is not directly comparable to outcome-based NNS estimates from established screening programs, it is expected to correspond to a downstream NNS to prevent one clinical event of approximately 100–150, assuming that detected abnormalities trigger evidence-based treatment with numbers needed to treat of around 20–30. Against this background, and considering that accepted screening programs often operate with substantially higher outcome-based NNS values, an NNS ≤ 5 was considered a clinically relevant benchmark for this pilot study [20].

### Exploratory outcomes

Exploratory outcomes comprise rates of GP consultation and medication changes, diagnostic metrics of multiparametric testing and individual test categories. Diagnostic metrics are assessed including sensitivity, specificity, positive predictive value (PPV), and negative predictive value (NPV). To further assess feasibility from participant perspective, the theoretical framework of acceptability (TFA Questionnaire) is used with the outcome of a median Likert-scale score of ≥ 4 for each item [21].

In addition, pharmacy-level data will be collected to describe heterogeneity across implementation sites and to support interpretation of pharmacy-level differences in feasibility and screening yield. These data will be separated into baseline pharmacy characteristics, implementation process metrics and recruitment process evaluation (Supplementary Table 2). Baseline pharmacy characteristics include staff size, number of pharmacists, pharmacy setting, approximate number of customers per day, opening hours, Saturday opening, status as the only pharmacy in town, prior experience with point-of-care testing or other structured pharmaceutical services, availability of a dedicated consultation area, availability of customer restroom facilities, stable internet access in the testing area, and existing collaboration with local general practitioners.

Pharmacy-level implementation process metrics will include the number of recruited participants per pharmacy, recruitment rate, recruitment pathway, sex and age distribution of recruited participants, inclusion criteria distribution, estimated time required per screening, staff group conducting the screening, technical issues, staff shortages during the recruitment period, perceived documentation burden, workflow compatibility, and main barriers to recruitment and screening completion. Furthermore, a recruitment process evaluation will assess factors influencing patient recruitment. These variables will be analyzed descriptively to explore heterogeneity across participating pharmacies and to inform the design of future larger-scale studies.

### Inclusion and exclusion criteria

Eligible participants are adults aged 40 years or older who visit a participating pharmacy and meet at least one of the criteria summarized in Table 1. Recruitment of the pharmacies will be done sequentially over a 3-month period, starting from 1 April 2026. Each pharmacy will have 6 months to enroll 50 customers in the study.

**Table 1:** Inclusion and exclusion criteria

Inclusion criteria	Exclusion criteria
Age ≥ 40 **and**	Participation in another study
Established regular GP **and**	Inability to give informed consent
Active smoking **or**	
Prescription of antihypertensive, antidiabetic, or lipid-lowering medication **or**	
Obesity (BMI ≥ 30 kg/m²; waist circumference ≥ 88 cm for women or ≥ 102 cm for men)	

### Procedures, data source and collection

#### Participant selection

Pharmacy customers will either be approached during a regular visit or contacted in advance, if they have authorized their pharmacy to inform them of health improvement projects. Potential participants based on the identification of risk medications will be identified via the pharmacy’s dispensing software. These medications will be pre-labeled within the internal inventory system, prompting a targeted invitation to the screening. A list of the drugs that are considered for the inclusion criteria is provided in Supplementary Table 3. Additionally, individuals may proactively ask for inclusion if they self-report to be active smokers, have a body mass index (BMI) ≥ 30 kg/m², or increased waist circumference (≥ 88 cm for women and ≥ 102 cm for men). Participating pharmacies will be instructed to aim for an approximately equal distribution of male and female participants.

The primary aim of this pilot implementation study is not to generate a population-representative prevalence estimate, but to evaluate whether a low-threshold CKM screening pathway can be implemented in community pharmacies with acceptable workflow burden. Therefore, pharmacies are allowed flexibility in recruitment as long as participants meet predefined eligibility criteria. This design is intended to preserve feasibility and external validity under routine pharmacy conditions. More restrictive recruitment procedures, such as strict consecutive sampling or quota-based recruitment, may reduce selection bias but would also increase workload and could impair recruitment, completion rates, and pharmacy engagement. To address selection bias, study monitoring will be implemented to improve balanced recruitment. Participating pharmacies will receive feedback after the inclusion of 25 participants. In addition, feedback will be provided if a male-to-female ratio of 3:1 is reached.

#### Implementation

An encompassing on-boarding concept will be applied to optimize implementation of the multi-parametric screening in pharmacies. This will include on-site training on the general project workflow, the use of the PoC tests and correct documentation. Training will be provided by the study team and PoC-device distributor Medic-SH. Informational videos and hand-outs demonstrating the correct application of the PoC tests will be made available to the pharmacies and participants. Technical support provided by the study center will be available to pharmacists and participants throughout the entire duration of the study. The screening is conceptually based on established and reimbursed clinical pharmacy services. Within APOSCREEN-1, community pharmacies receive a reimbursement of €50 per completed screening [22].

### Data source and collection at screening

In the initial visit, written informed consent will be obtained by the pharmacist and relevant data will be documented using an electronic case report form (eCRF). Table 2 displays the data collected at different time points. Subsequently, participants undergo standardized PoC assessments including albuminuria, blood pressure, HbA1c and full lipid profile (total cholesterol, non-high-density lipoprotein (non-HDL-C) cholesterol, HDL cholesterol (HDL-C), low-density lipoprotein cholesterol (LDL-C) and triglycerides). In pharmacies equipped for on-site PoC testing of albuminuria and blood pressure, all tests will be conducted at the initial visit. Alternatively, patients will be provided with validated home blood pressure monitors and semi-quantitative albuminuria tests along with instructions for testing. Results will be assessed in a second appointment in the pharmacy, together with PoC testing for HbA1c and lipid profile.

**Table 2:** Data collected within the eCRF

Category	Data collected		
	At Screening	At Confirmatory Testing	At Follow-Up
Demographic data	• age • gender • postal code • educational level • employment status • type of health insurance		
History	• currently prescribed medication list • pre-existing medical conditions • family history of cardiovascular disease • smoking status • last participation in a health check-up		• medication • visits to the general practitioner after screening findings and therapy • changes from the GP visit • satisfaction with screening / self-assessed benefit of the project
Clinical	• home blood pressure (3x) • body weight • body height • calculated BMI • waist circumference	• 24-hour blood pressure	
Laboratory	• HbA1c • lipid profile (total cholesterol, non-HDL-cholesterol, calculated LDL, HDL, triglycerides) • albuminuria (albuminuria test or urine-albumin-creatinine-ratio)	• HbA1c • fasting plasma glucose • lipid profile (total cholesterol, LDL, HDL, triglycerides, lipoprotein(a)) • creatinine, cystatin c • urine-albumin-creatinine-ratio	

GP, General Practitioner; HDL, high-density lipoprotein; LDL, low-density lipoprotein, BMI, body mass index

### Methods and equipment used for screening

All invasive PoC tests are conducted by pharmacists who have received structured training and technical guidance from the study team. Blood pressure is recorded using a validated automatic device and participants will take three consecutive seated measurements after a five-minute rest period.

For screening the following equipment will be used:

1. Home-based albuminuria testing: Siegmund Care GmbH, Oberottmarshausen, Germany.
2. Pharmacy based albuminuria testing (UACR): Afinion 2, Abbott Rapid Diagnostics Germany GmbH, Köln, Germany.
3. HbA1c: A1CNow+, Medic-SH, Reinfeld, Germany.
4. LDL-C: PTS CardioCheck Plus, Medic-SH, Reinfeld, Germany.
5. Blood pressure: M2 Comfort, OMRON, Kyōto, Japan.

### Data transmission and analysis

The collected screening parameters are securely transmitted to the study center. The digital study platform, including the eCRF and data infrastructure, is provided by meditrova GmbH (Rostock, Germany). It is accessible via smartphone, tablet or personal computer through the internet and an application (*App*). After screening completion, a physician at the study center will review and analyze the data. Based on the results, patients are further stratified:

1. For unremarkable screening results a digital follow-up is conducted.
2. For positive screening results a confirmatory testing and digital follow-up is conducted.

### Confirmatory testing

Participants are invited to undergo confirmatory assessment if screening results meet at least one of the predefined thresholds: systolic blood pressure ≥ 140 mmHg and/or diastolic blood pressure ≥ 90 mmHg, positive urine albuminuria test (corresponding to an albuminuria ≥A2 and urine-albumin-creatinine-ratio of ≥ 30 mg/g), a non-HDL-C ≥ 5.7 mmol/l or HbA1c ≥ 5.7% for patients without and ≥ 8.5% for patients with known diabetes (Supplementary Figures S1-4 [23–26]). In case of critical values at screening, pharmacists will be guided by an emergency algorithm (Supplementary Figure S5).

Confirmatory testing will be performed at the study center to enable standardized reassessment. To ensure representative coverage of both urban and rural regions, participating pharmacies will be selected accordingly, allowing the study to capture regional heterogeneity in patient presentation and healthcare access. In this context, ten pharmacies located at greater geographical distance (> 30 km) from the study center will be included.

**Fig. 1:**
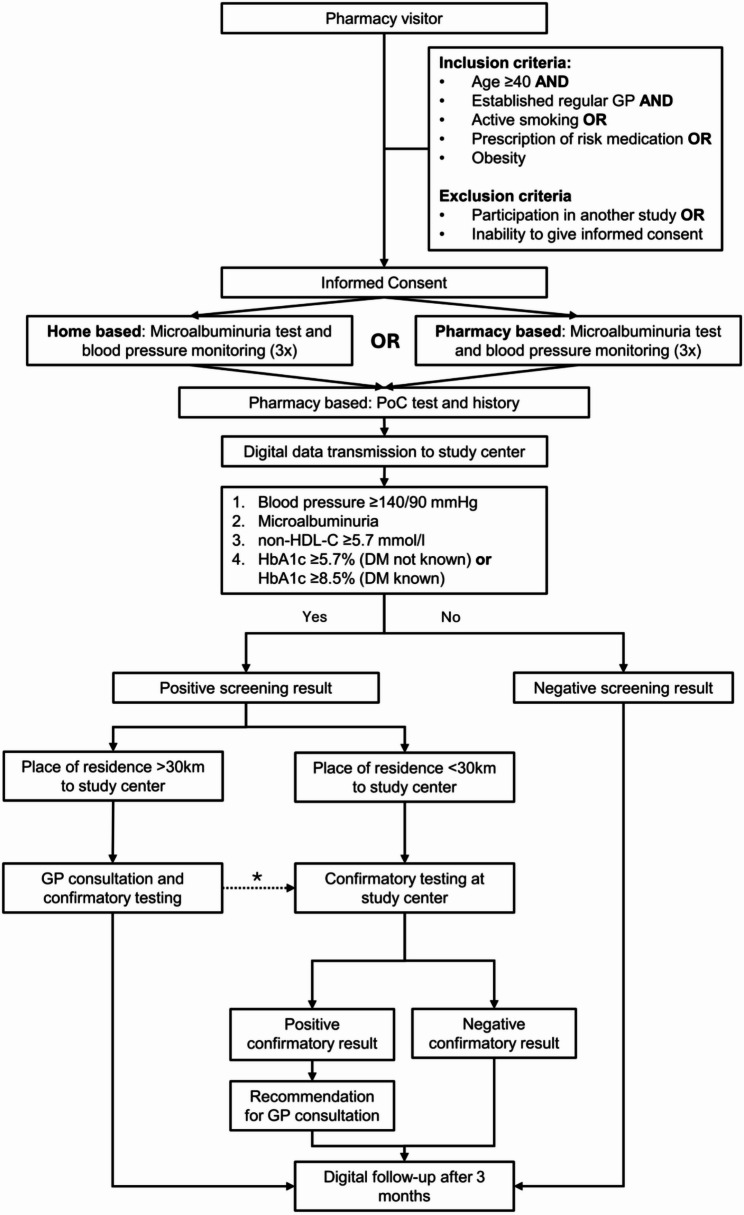
Flow chart of study recruitment, testing and follow-up. GP, General Practitioner; PoC-Test, Point-of-Care-Test; HDL, high-density lipoprotein; DM, Diabetes mellitus; Obesity defined as (BMI ≥30kg/m² or waist circumference ≥ 88cm for women or ≥ 102cm for men); * if confirmatory testing is not feasible at the GP, participants can conduct confirmatory testing at the study center

At the study center visit, all previously screened parameters will be reassessed using standardized laboratory methods. In addition, 24-hour ambulatory blood pressure monitoring (ABPM) or 7-day home-based blood pressure monitoring (HBPM) will be conducted. Blood measurement of creatinine, CRP and lipoprotein(a) will be taken for improved precision of the risk assessment. The patient pathway is visualized in Fig. 1.

### Patient risk report and digital follow-up

Based on medical history, screening results, and, if available, confirmatory testing outcomes, an individualized risk report based on current national and international guidelines is generated and made accessible for the participant through the digital study platform. The report will provide a structured summary of all relevant findings, with a particular focus on estimated cardiovascular risk (SCORE2, SCORE2-OP, SCORE2-Diabetes, SMART2-Score, depending on applicability) and risk of kidney replacement therapy (kidney failure risk equation), and will serve as a structured basis for follow-up consultations within the primary care system [27–30]. Participants with unremarkable screening results will receive a report providing general evidence-based recommendations for general CKM syndrome risk reduction.

### Data storage

Data will be pseudonymized and stored on servers of the study center for 10 years after study completion. Study investigators will have access to information for re-identification. Participants may withdraw consent at any time and request deletion of their data. All data shared for research will be anonymized.

### Sample size and statistical analysis

The planned sample size of 1000 participants allows for reliable estimation of key outcomes with 95% confidence intervals with an estimation error of approximately ± 5%. With an expected prevalence of 50% of risk factors and 10 pharmacies in the region of the study center, we expect 250 participants with indication for confirmatory testing at the study center. At an expected attrition of 20%, 200 participants should complete confirmatory testing. This allows for determination of false-positive rates and test specificity with acceptable precision (± 10%). Attrition in the GP-confirmatory testing arm is expected to be higher, but will be minimized through repeated digital or telephone prompts and, if necessary, invitation for testing at the study center. Statistical analysis will be descriptive, using frequency tables, means (± standard deviation), medians (± interquartile range) for Likert-Scale analysis, and proportions with 95% confidence intervals. Diagnostic metrics (sensitivity, specificity, PPV, NPV) will be determined. Sensitivity of the PoC test can be assessed based on the results from the confirmatory testing. Scale scores for implementation metrics will be calculated as the mean of the respective items, with higher values indicating greater agreement and lower values indicating greater disagreement.

Feasibility and clinical outcomes will be summarized overall and, where appropriate, descriptively at pharmacy level. Pharmacy-level summaries will include recruitment numbers, screening completion rates, confirmatory testing uptake, diagnostic yield, and implementation scores. Baseline pharmacy characteristics will be used to contextualize variation across pharmacies, whereas pharmacy-level implementation process metrics will be interpreted as outcomes of the implementation process. Given the limited number of participating pharmacies, these analyses will be exploratory and hypothesis-generating. Confirmatory inference regarding pharmacy-level predictors is not planned.

Implementation outcomes will be analyzed separately for each implementation instrument and domain. AIM, FIM, and NoMad assess conceptually distinct implementation constructs, including acceptability, feasibility, and normalization/sustainability. Therefore, no combined implementation score or omnibus test will be used. For each domain, median Likert-scale scores and interquartile ranges will be reported. In addition, one-sided one-sample Wilcoxon signed-rank tests will be used to assess whether domain scores exceed the neutral midpoint of the scale, using the null hypothesis that the median domain score is ≤ 3. Given the exploratory nature of this pilot implementation study and the domain-specific interpretation of implementation outcomes, no formal multiplicity adjustment is planned. Results will be interpreted separately by domain and in the context of the overall implementation pattern.

The unadjusted NNS will be calculated as the number of participants with completed screening divided by the number of participants with at least one previously unknown or insufficiently managed CKM risk factor identified during screening. For primary outcome, a pre-specified PPV-adjusted NNS will be calculated using the positive predictive value estimated among screen-positive participants undergoing confirmatory testing at the study center. Participants without available confirmatory testing will not be included in the estimation of the PPV. Stratified NNS according to pharmacy-prompted or self-referral recruitment will be further reported.

### Missing data

Missing data will be reported for each variable individually and will be transparently documented for all variables included in the analysis, and reported as part of the descriptive results. Within the electronic case report form (eCRF), participants are given the option to select an “unknown” category where applicable, thereby allowing explicit capture of non-available responses at the point of data entry. Responses documented as “unknown” will be reported descriptively and treated as missing for analyses requiring classification of a risk factor as previously known, unknown, controlled, or insufficiently managed.

No formal imputation of missing data is planned given the exploratory character of this pilot implementation study.

Participants without available confirmatory testing for the respective parameter will not be included in parameter-specific PPV or diagnostic accuracy calculations. Uptake of confirmatory testing and missing confirmatory results will be reported descriptively. For implementation questionnaires, domain scores will be calculated only if sufficient item-level data are available for the respective domain. Otherwise, the domain score will be treated as missing. No item-level imputation is planned.

For the participant-level completion rate, participants who provide informed consent and start the screening procedure will remain in the denominator; participants with incomplete subsequent screening procedures will be counted as not completed. For the primary NNS analysis, only participants with completed screening procedures will be included in the denominator. Participants with incomplete screening procedures will therefore not contribute to the NNS analysis, but their number will be reported through the completion-rate outcome.

## Discussion

The burden of CKM syndrome remains a major, yet increasing, challenge for healthcare systems worldwide [31]. In Germany, one of the key reasons are the low participation rates of population-based screening programs that aim to identify CKM syndrome risk factors [8]. Therefore, innovation for a more effective prevention is urgently needed.

The APOSCREEN-1 study investigates the potential of community pharmacies to help redefine preventive care strategies. A comprehensive dataset will be generated, capturing key metrics such as patient reach, acceptance and completion rates, as well as medical outcomes and pharmacist-reported applicability of the screening workflow. On this basis, primary outcomes were chosen to evaluate the overall applicability of the screening in community pharmacies, encompassing implementability, feasibility, and potential clinical benefit, which are key considerations for future scalability.

Our multi-parametric screening-approach will triage patients for further testing who are at significant risk for cardiovascular events or kidney disease while avoiding an overly low threshold for further work-up in order to maintain health care resource feasibility. Through this, the study is designed to address three central challenges of the German healthcare system:

First, a significant proportion of individuals remain excluded from prevention strategies due to logistical, social, and systemic barriers [32]. Primary care relies on voluntary check-ups, which limits general practitioners (GP) ability to reach those who do not attend medical practices. In contrast, the APOSCREEN-1 study proposes a widely accessible and frequently used care setting. By collecting screening data, the study will evaluate whether low-threshold screening in pharmacies may enhance access to screening.

Second, the incidence of CKM syndrome risk factors is increasing and a considerable number of medically underserved individuals can reasonably be assumed. Based on the obtained data, the APOSCREEN-1 study will enable the assessment of: (I) the prevalence of pathological findings in this at-risk population, (II) the proportion of individuals whose CKM syndrome risk factors remain untreated or insufficiently managed and (III) the number needed to screen for a previously unobserved risk factor (NNS) along with the positive predictive value of the PoC testing. As an exploratory endpoint, the study will assess whether pathological findings translate into changes in prescription and management by primary care providers.

Third, Germany faces an aging population with limited personnel resources [33–35]. Scalable, decentralized models are warranted. Community pharmacies are professionally staffed locations and well-positioned to support preventive care. Their increasing responsibilities in patient education, vaccination, and the use of diagnostic tools underline the health-serving potential. Feasibility analysis will provide insight into the applicability of a large-scale implementation and its challenges.

### Limitations

Participants receive an individualized, guideline-based risk report and recommendation tailored to the participant’s screening, nevertheless therapy induction relies on GP consultation. Currently, no universally accepted guideline-based NNS threshold exists for clinically meaningful detection efficiency. Instead, screening frameworks emphasize overall net benefit; for example, the United States Preventive Services Task Force does not use a fixed number needed to treat, screen, or harm as a decision threshold [20]. Therefore the NNS used reflects only a pragmatic approach. Due to the recruitment process, residual selection bias cannot be excluded, but will be mitigated through regular study monitoring during the recruitment phase. In addition, healthy volunteer bias may limit generalizability of patient-level findings, as participants opting into screening might be more health-conscious than the general population. Further, pharmacy-level selection bias cannot be excluded, as participating pharmacies were self-selected and may not be representative of all community pharmacies.

If APOSCREEN-1 demonstrates feasibility and sufficient diagnostic yield, a larger effectiveness study will be warranted. Such a follow-up trial could evaluate a scaled pharmacy-based screening approach in a more representative sample of pharmacies across Schleswig-Holstein and potentially additional German states, using health-insurance claims data to compare screened individuals with matched non-participants. Key outcomes should include GP-confirmed diagnosis of previously unknown or insufficiently managed CKM risk factors, initiation or intensification of guideline-based therapy, and, with longer follow-up, CKM-related hospitalizations or death.

In conclusion, APOSCREEN-1 addresses a timely and structural need to strengthen early CKM syndrome risk identification by leveraging community pharmacies. The findings will be essential to guide the development of large-scale screening efforts and policy discussions around integrating pharmacies into national prevention frameworks.

## Electronic Supplementary Material

Below is the link to the electronic supplementary material.

Supplementary Material 1

Supplementary Material 2

Supplementary Material 3

Supplementary Material 4

Supplementary Material 5

Supplementary Material 6

## Data Availability

Data will be shared with researchers who provide a methodically sound proposal. Individual deidentified data collected during the trial will be made accessible. Proposals should be directed to the corresponding author. Data requestors must sign a data access agreement to gain access. Data will be shared to achieve the aims in the approved proposal.

## Abbreviations

CKD: Chronic kidney disease
CKM syndrome: Cardiovascular–kidney–metabolic syndrome
CVD: Cardiovascular disease
eCRF: Electronic case report form
EU: European Union
GDP: Gross domestic product
GP: General practitioner
BMI: Body mass index
HBPM: Home blood pressure monitoring
HDL: C–high–density lipoprotein cholesterol
LDL: C–low–density lipoprotein cholesterol
NNS: Number needed to screen
NPV: Negative predictive value
PPV: Positive predictive value
PoC: Point–of–care
RASi: Renin–angiotensin–aldosterone system inhibitors
SGLT2i: Sodium–glucose cotransporter–2 inhibitor
SD: Standard deviation
TC: Total cholesterol
UACR: Urine albumin–creatinine ratio
