# Supplementary figures and images for: APOSCREEN-1 – a prospective, single-arm clinical trial for the implementation of a pharmacy-based screening for cardiovascular-kidney-metabolic risk factors in Schleswig-Holstein
Source: BMC Nephrol. 2026 Jun 5;27:357. doi: 10.1186/s12882-026-05090-x (PMC13244955; doi:10.1186/s12882-026-05090-x)

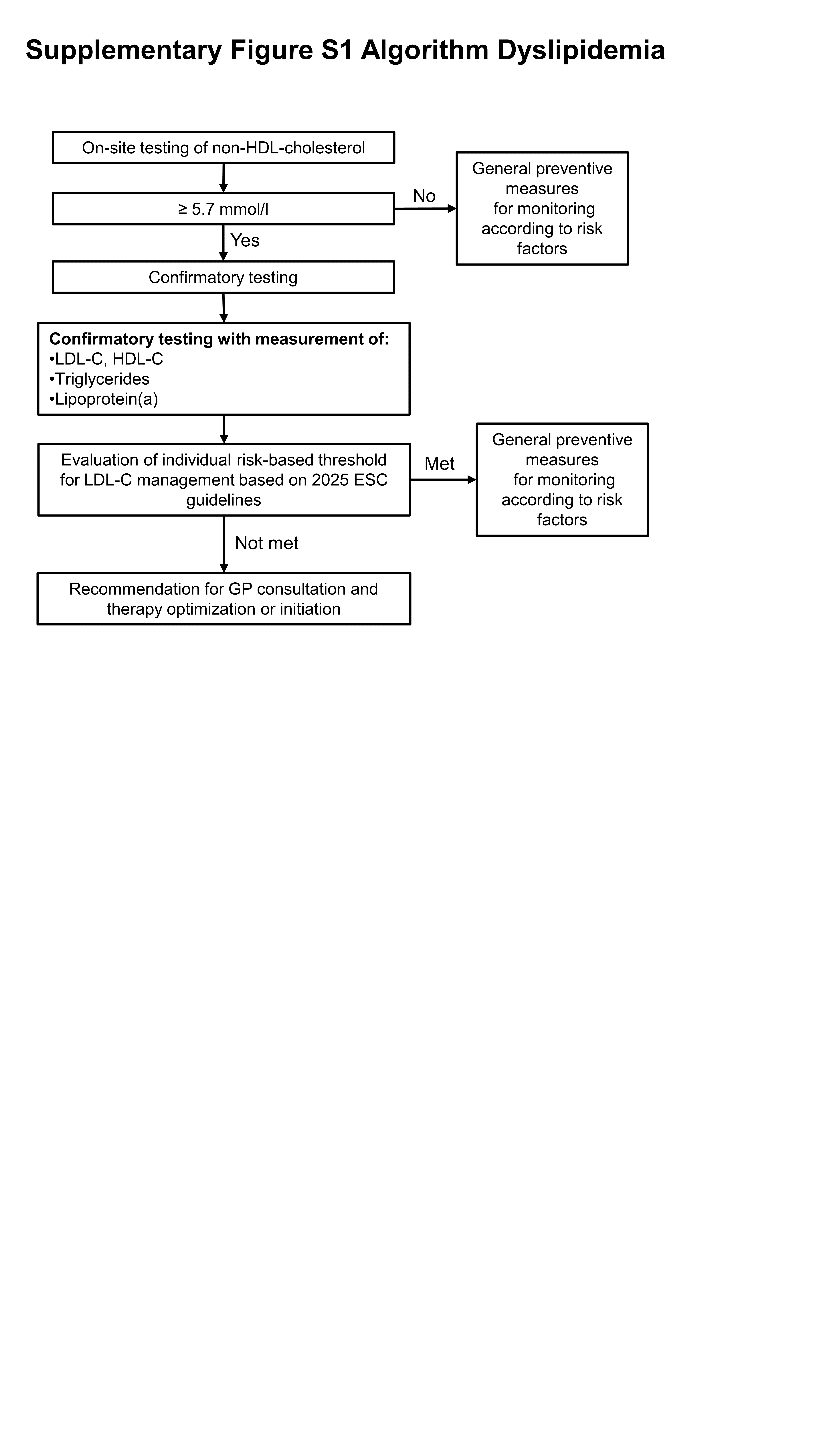


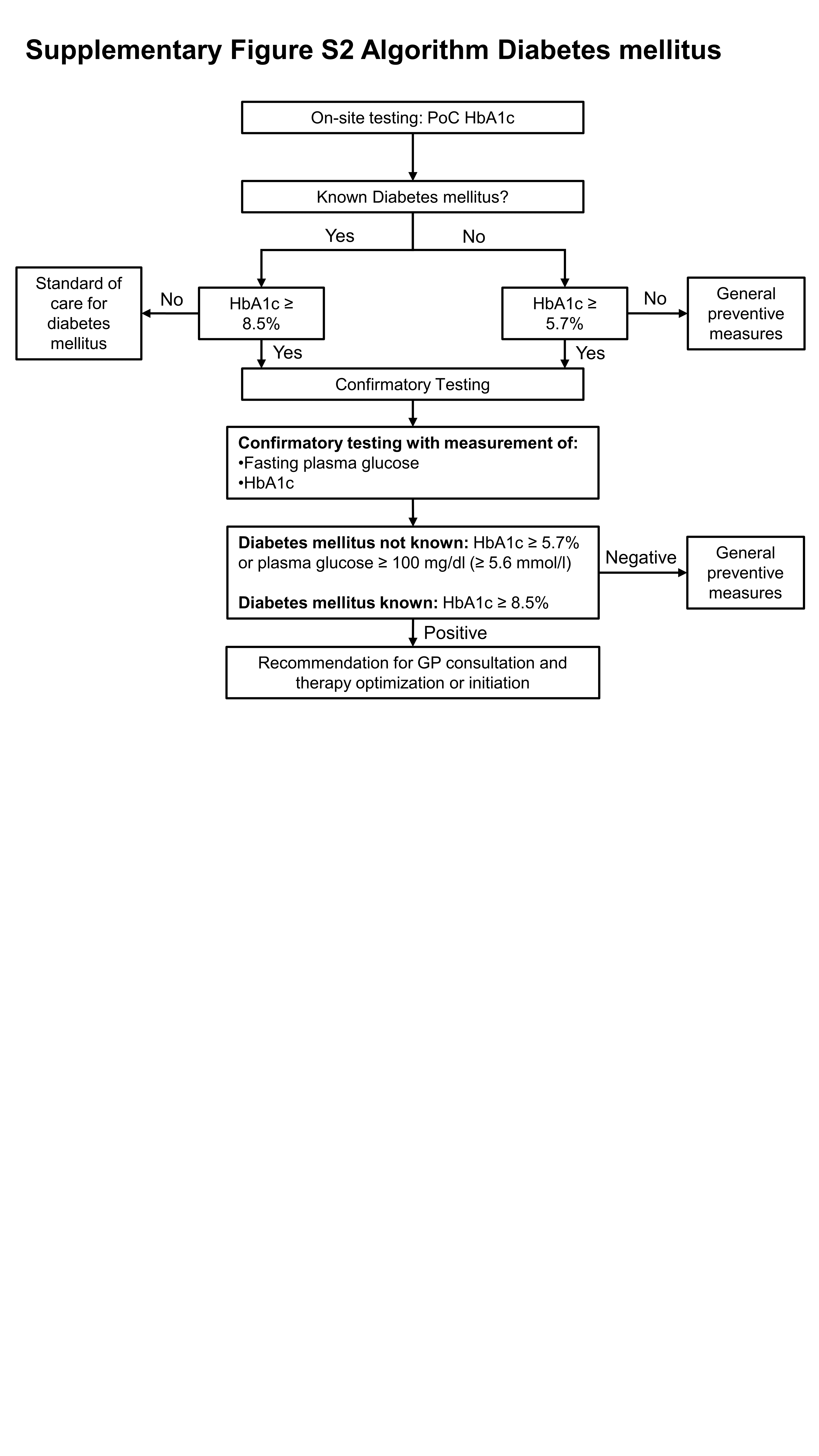


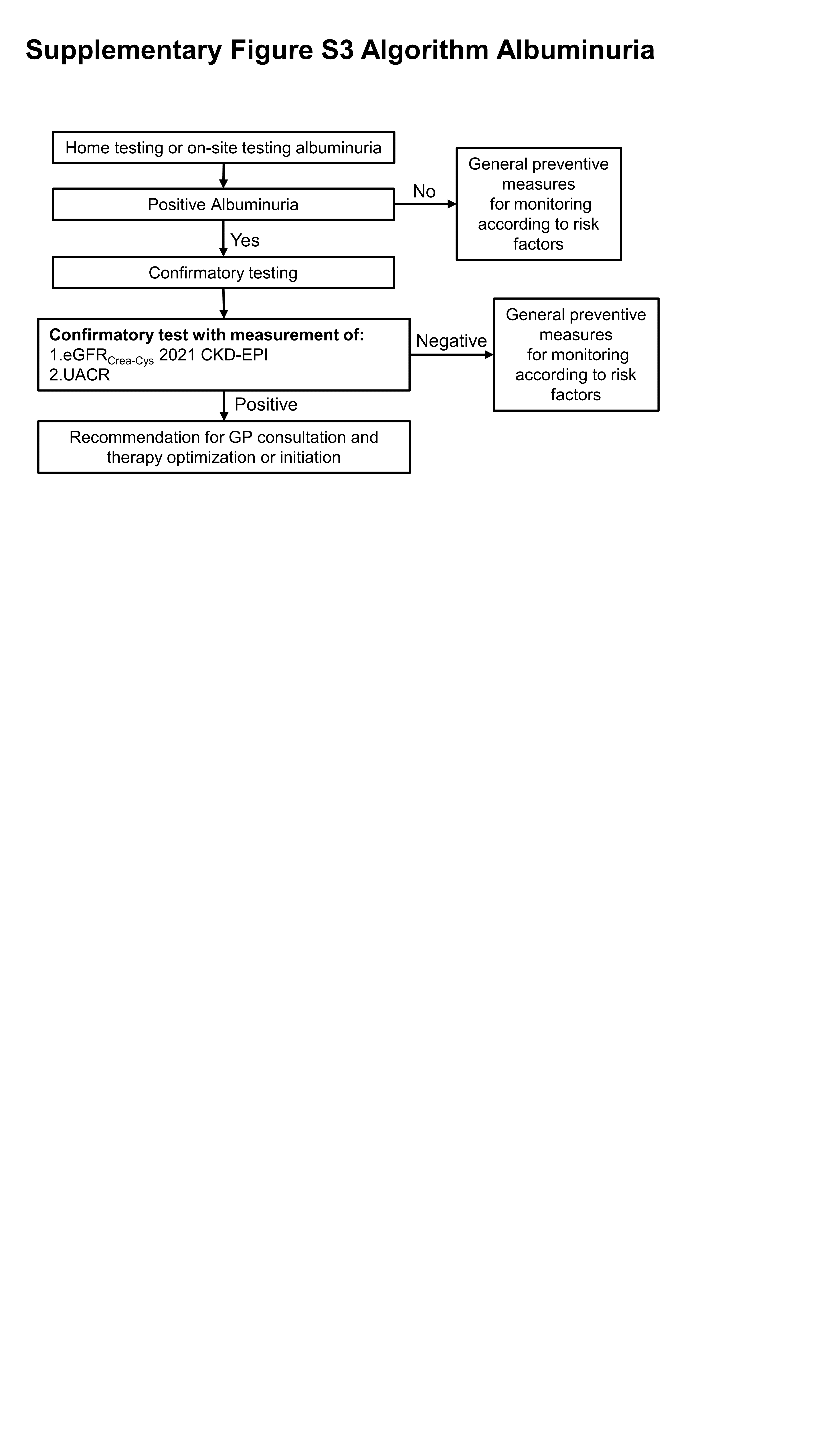


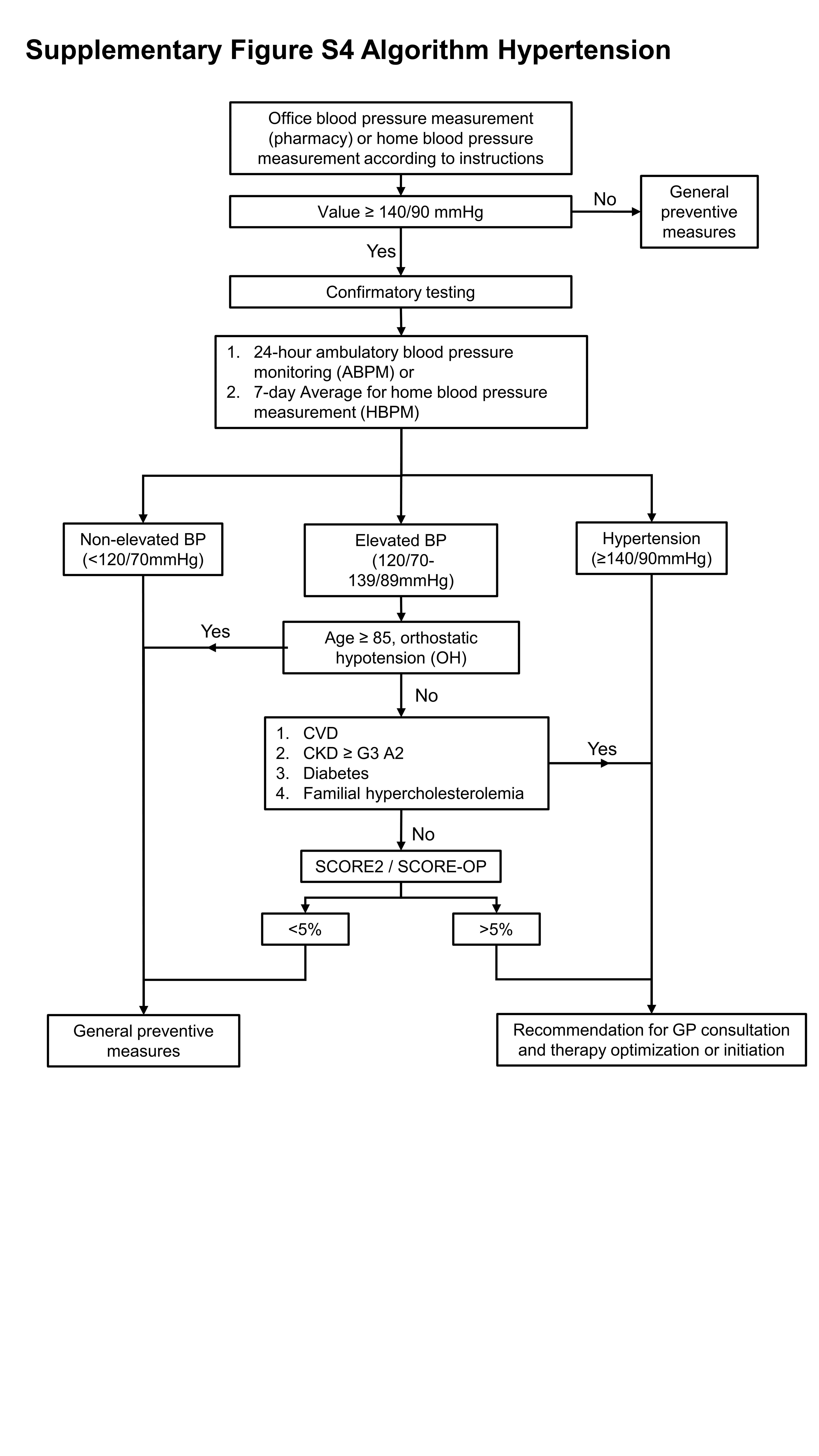


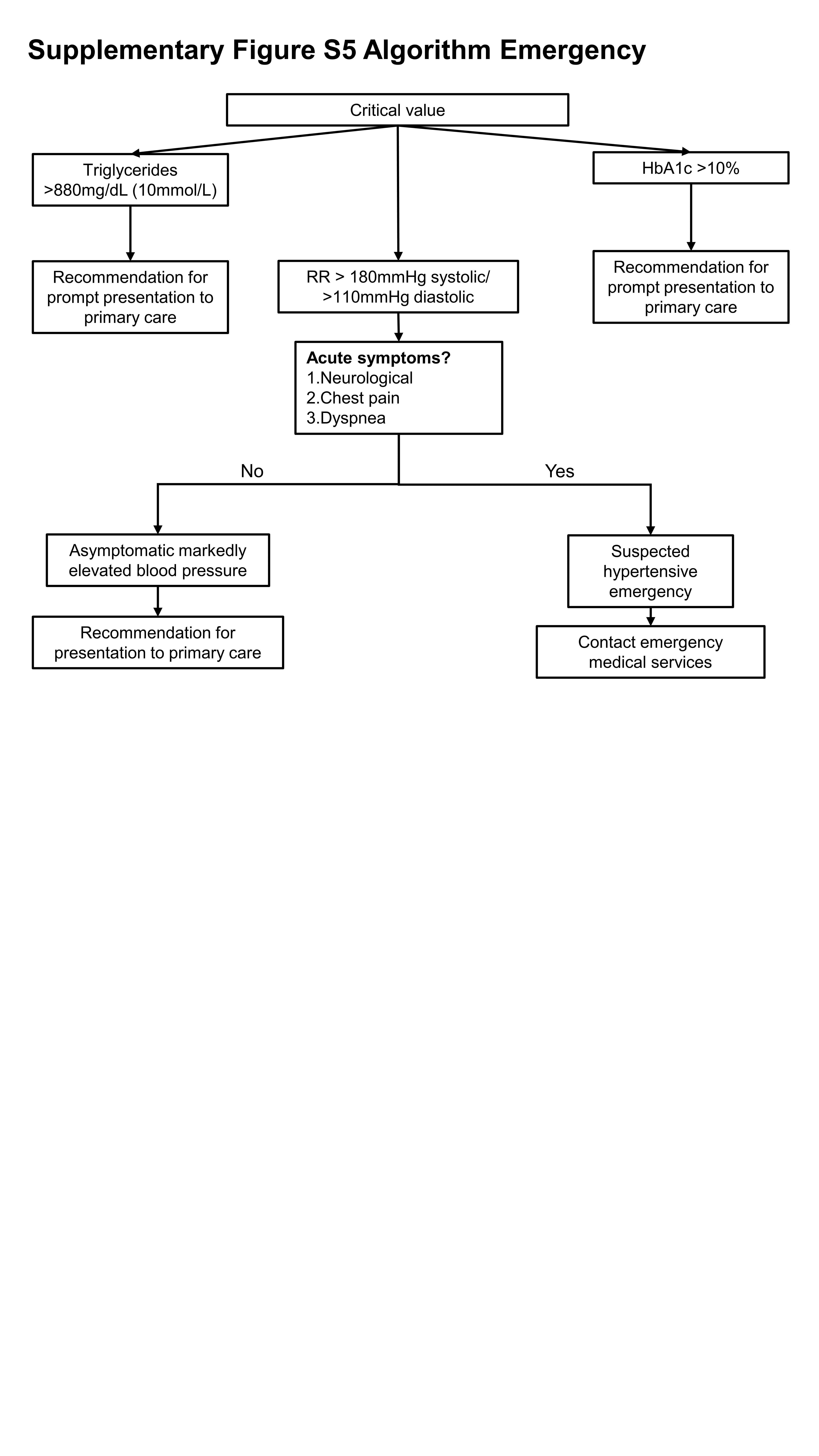

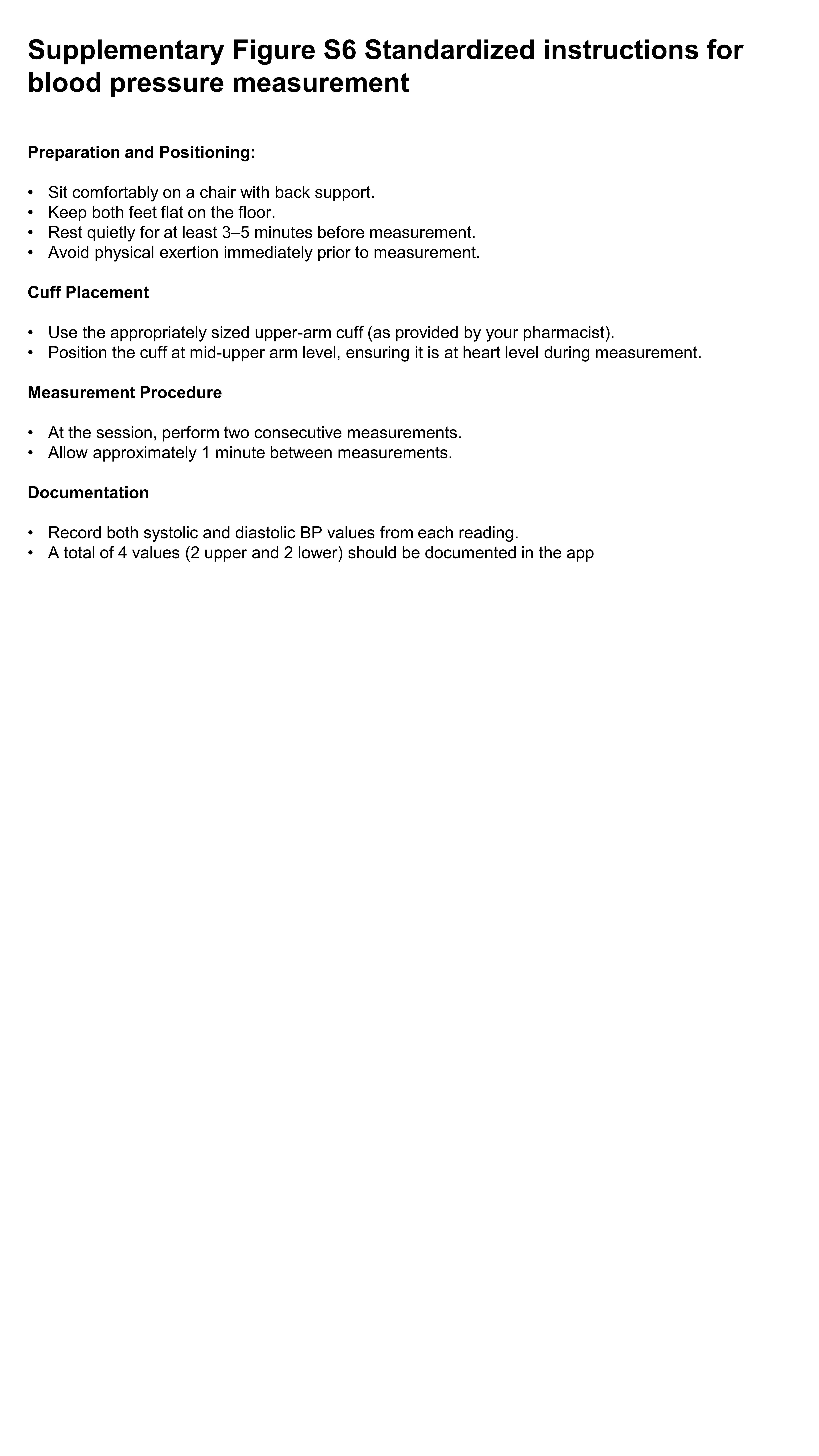

Supplement: Supplementary file 1 — Supplementary Material 1 [file 12882_2026_5090_MOESM1_ESM.docx]
