# Supplementary material for: APOSCREEN-1 – a prospective, single-arm clinical trial for the implementation of a pharmacy-based screening for cardiovascular-kidney-metabolic risk factors in Schleswig-Holstein
Source: BMC Nephrol. 2026 Jun 5;27:357. doi: 10.1186/s12882-026-05090-x (PMC13244955; doi:10.1186/s12882-026-05090-x)
Supplement: Supplementary file 2 — Supplementary Material 2 [file 12882_2026_5090_MOESM2_ESM.docx]

**Supplementary Table 1: Measurement of implementation outcomes for evaluation of the pharmacy-perspective**

**Acceptability of Intervention Measure (AIM)**

|  | Completely disagree | Disagree | Neither agree nor disagree | Agree | Completely agree |
| --- | --- | --- | --- | --- | --- |
| 1. APOSCREEN meets my approval. | ➀ | ➁ | ➂ | ➃ | ➄ |
| 2. APOSCREEN is appealing to me. | ➀ | ➁ | ➂ | ➃ | ➄ |
| 3. I like APOSCREEN | ➀ | ➁ | ➂ | ➃ | ➄ |
| 4. I welcome APOSCREEN. | ➀ | ➁ | ➂ | ➃ | ➄ |

**Feasibility of Intervention Measure (FIM)**

|  | Completely disagree | Disagree | Neither agree nor disagree | Agree | Completely agree |
| --- | --- | --- | --- | --- | --- |
| 1. APOSCREEN seems implementable in routine business life. | ➀ | ➁ | ➂ | ➃ | ➄ |
| 2. APOSCREEN seems possible in our pharmacy | ➀ | ➁ | ➂ | ➃ | ➄ |
| 3. APOSCREEN seems doable. | ➀ | ➁ | ➂ | ➃ | ➄ |
| 4. APOSCREEN seems easy to use. | ➀ | ➁ | ➂ | ➃ | ➄ |
