# Supplementary material for: APOSCREEN-1 – a prospective, single-arm clinical trial for the implementation of a pharmacy-based screening for cardiovascular-kidney-metabolic risk factors in Schleswig-Holstein
Source: BMC Nephrol. 2026 Jun 5;27:357. doi: 10.1186/s12882-026-05090-x (PMC13244955; doi:10.1186/s12882-026-05090-x)
Supplement: Supplementary file 3 — Supplementary Material 3 [file 12882_2026_5090_MOESM3_ESM.docx]

**Supplementary Table 2: Questionnaire for pharmacists for baseline characteristics, workload and screening metrics**

| **Question** | **Answer Options** |
| --- | --- |
| **Baseline characteristics** | |
| What is the size of the population served by your pharmacy (catchment area)? | <3000, 3000-5000, 5000-10.000, >10.000 |
| How many pharmacists are employed at your pharmacy? | Absolute number |
| How many pharmacy technicians are employed at your pharmacy? | Absolute number |
| How many pharmaceutical commercial assistants are employed at your pharmacy? | Absolute number |
| Is your pharmacy the only pharmacy in the town/community? | Yes/ No; absolute number of pharmacies |
| Opening hours; Open on Saturdays | Opening hours |
| How would you describe the location of your pharmacy? | Urban (City), small town, rural |
| Do you have prior experience with point-of-care testing or structured pharmaceutical services? | Yes/ No |
| Was point-of-care testing already part of your routine daily practice before participation in this project? | Yes/ No |
| Is a dedicated consultation area available for patient counselling or testing? | Yes/ No |
| Is a customer restroom available in your pharmacy? | Yes/ No |
| Is a stable internet connection available in the testing area? | Yes/ No |
| Is there an existing collaboration with local general practitioners (GPs)? | Yes/ No |
| **Workload and screening metrics** | |
| How long did it take to reach the recruitment target in your pharmacy? | <1 month, 1–3 months, 3 months, 3-6 months, not reached |
| How much time did the project implementation take? | <2 hours, 2-4 hours, >4 hours |
| How would you rate the overall workload of the screening process for your team? | Very low, Low, Moderate, High, Very high |
| How many patients visit your pharmacy per week on average? | <400, 400-800, 800-1200, >1200 |
| How many patients were screened per month on average during the project? | <5, 5–9, 10–19, 20–29, ≥30 |
| What proportion of approached patients agreed to participate in the screening? | <10%, 10-25%, 25-50%, 50-75%, >75% |
| How much time did the screening process take on average per patient (including testing, history taking, informed consent, documentation) for you and your team? | <20 minutes, 20-30 minutes, 30-45 minutes, >45 minutes |
| Which staff group primarily conducted the screening? | Pharmacist, Pharmacy technician, Pharmaceutical commercial assistant, Shared between staff groups |
| Did staff shortages occur during routine daily pharmacy operations in the recruitment period? | Never, rarely, sometimes, often, very often |
| Perceived documentation burden associated with the screening process: | <10min, 10-20min, 20-30min, >30min |
| How well was the screening workflow compatible with routine pharmacy operations? | Free text response |
| What were the main barriers to screening completion from the pharmacy perspective? | Free text response |
| **Recruitment process evaluation** | |
| What were the main barriers to patient recruitment (approach and enrolment)? | Free text response |
| What factors facilitated patient recruitment? | Free text response |
| Which part of the recruitment process was most challenging? | Patient approach, explaining study, consent process, PoC-testing, documentation, other (free text response) |
| Which patient groups were more likely to participate? | Regular customers, walk-in customers, elderly patients (>60, younger patients (40-60), no clear pattern, other (free text response)__ |
| Which patient groups were more difficult to recruit? | Free text response |
| Main reasons for patient refusal: | Lack of time, no interest, no perceived benefit, privacy concerns, no perceived need, other (free text response)____ |
| Were refusals time-dependent? | Yes/ No If yes: Morning, Midday, Afternoon, Evening |
| Most effective point of patient approach: | Prescription dispensing, consultation, checkout/payment, waiting time, no specific point, other (free text response) |
| Recruitment was mainly: | Proactive (pharmacy-initiated), reactive (patient-driven interaction), both |
| Who primarily initiated recruitment? | Pharmacist, PTA, PKA, shared |
| Did workload peaks affect recruitment success? | Never, rarely, sometimes, often, very often |
| What changes would improve recruitment? | Free text response |
| Where there perceived sex differences in interest in the study? | Yes, primarily male participants were more interested, primarily female participants were more interested, no difference observed |
