# Supplementary material for: APOSCREEN-1 – a prospective, single-arm clinical trial for the implementation of a pharmacy-based screening for cardiovascular-kidney-metabolic risk factors in Schleswig-Holstein
Source: BMC Nephrol. 2026 Jun 5;27:357. doi: 10.1186/s12882-026-05090-x (PMC13244955; doi:10.1186/s12882-026-05090-x)
Supplement: Supplementary file 5 — Supplementary Material 5 [file 12882_2026_5090_MOESM5_ESM.docx]

**Survey Instructions**

This survey asks questions about the implementation of **APOSCREEN**. We understand that people involved with **APOSCREEN** have different roles, and that people may have more than one role.

The survey is in 3 parts. Part A asks some brief questions about yourself and your role. Part B includes three general questions about **APOSCREEN**, Part C contains a set of more detailed questions about **APOSCREEN**. For each statement in Part C, there is the option to agree or disagree with what is being asked **(OPTION A).** However, if you feel that the statement is not relevant to you, there are also options to tell us why **(OPTION B).**

Please take the time to decide which answer **best suits your experience for each statement and tick the appropriate circle**

| **Part A: About yourself** | | | | | | | | | | | |  |  |
| --- | --- | --- | --- | --- | --- | --- | --- | --- | --- | --- | --- | --- | --- |
| 1. **How many years have you worked for this pharmacy? *(If your Trust has merged with another or changed its name, please include in your answer all the time you have worked with this Trust and its predecessors)*** | | | | | | | | | | | | | |
|  | | Less than one year |  | 1-2 years |  | 3-5 years |  | 6-10 years |  | 11-15 years |  | More than 15 years |  |
| 1. **How would you describe your professional job category?** | | | | | | | | | | | | | |

|  |
| --- |

| **Part B: General questions about the intervention** |
| --- |
|  |

| When you use APOSCREEN, how familiar does it feel? | | | | | | | | | | | | |
| --- | --- | --- | --- | --- | --- | --- | --- | --- | --- | --- | --- | --- |
| Still feels very new | | | |  | | | | | **Feels completely familiar** | | | |
|  | | | |  | | | | |  | | | |
| 0 | 1 | 2 | 3 | | 4 | 5 | 6 | 7 | | 8 | 9 | 10 |
| Do you feel APOSCREEN is currently a normal part of your work? | | | | | | | | | | | | |
| Not at all | | | | **Somewhat** | | | | | **Completely** | | | |
|  | | | |  | | | | |  | | | |
| 0 | 1 | 2 | 3 | | 4 | 5 | 6 | 7 | | 8 | 9 | 10 |
| Do you feel APOSCREEN will become a normal part of your work? | | | | | | | | | | | | |
|  | | | | | | | | | | | | |
| Not at all | | | | **Somewhat** | | | | | **Completely** | | | |
|  | | | |  | | | | |  | | | |
| 0 | 1 | 2 | 3 | | 4 | 5 | 6 | 7 | | 8 | 9 | 10 |

| **Part C: Detailed questions about the intervention** |
| --- |

**For each statement please select an answer that best suits your experience using Option A. If the statement is not relevant to you please select an answer from Option B.**

|  | | **Option A** | | | | |  | **Option B** | | |
| --- | --- | --- | --- | --- | --- | --- | --- | --- | --- | --- |
| **Section C1** | | **Strongly Agree** | **Agree** | **Neither agree nor disagree** | **Disagree** | **Strongly disagree** |  | **Not relevant to my role** | **Not relevant at this stage** | **Not relevant to the intervention** |
| **1.** | **I can see how APOSCREEN differs from usual ways of working** |  |  |  |  |  |  |  |  |  |
| **2.** | **Staff in my organisation have a shared understanding of the purpose of APOSCREEN** |  |  |  |  |  |  |  |  |  |
| **3.** | **I understand how APOSCREEN affects the nature of my own work** |  |  |  |  |  |  |  |  |  |
| **4.** | **I can see the potential value of APOSCREEN for my work** |  |  |  |  |  |  |  |  |  |

**For each statement please select an answer that best suits your experience using Option A. If the statement is not relevant to you please select an answer from Option B.**

|  | |  |  | **Option A** | |  |  | **Option B** | | |
| --- | --- | --- | --- | --- | --- | --- | --- | --- | --- | --- |
| **Section C2** | | **Strongly Agree** | **Agree** | **Neither agree nor disagree** | **Disagree** | **Strongly disagree** |  | **Not relevant to my role** | **Not relevant at this stage** | **Not relevant to the intervention** |
| **1.** | **There are key people who drive APOSCREEN forward and get others involved** |  |  |  |  |  |  |  |  |  |
| **2.** | **I believe that participating in APOSCREEN is a legitimate part of my role** |  |  |  |  |  |  |  |  |  |
| **3.** | **I’m open to working with colleagues in new ways to use APOSCREEN** |  |  |  |  |  |  |  |  |  |
| **4.** | **I will continue to support APOSCREEN** |  |  |  |  |  |  |  |  |  |

**For each statement please select an answer that best suits your experience using Option A. If the statement is not relevant to you please select an answer from Option B.**

|  | | **Option A** | | | | |  | **Option B** | | |
| --- | --- | --- | --- | --- | --- | --- | --- | --- | --- | --- |
| **Section C3** | | **Strongly Agree** | **Agree** | **Neither agree nor disagree** | **Disagree** | **Strongly disagree** |  | **Not relevant to my role** | **Not relevant at this stage** | **Not relevant to the intervention** |
| **1.** | **I can easily integrate APOSCREEN into my existing work** |  |  |  |  |  |  |  |  |  |
| **2.** | **APOSCREEN disrupts working relationships** |  |  |  |  |  |  |  |  |  |
| **3.** | **I have confidence in other people’s ability to use APOSCREEN** |  |  |  |  |  |  |  |  |  |
| **4.** | **Work is assigned to those with skills appropriate to APOSCREEN** |  |  |  |  |  |  |  |  |  |
| **5.** | **Sufficient training is provided to enable staff to implement APOSCREEN** |  |  |  |  |  |  |  |  |  |
| **6.** | **Sufficient resources are available to support APOSCREEN** |  |  |  |  |  |  |  |  |  |
| **7.** | **Management adequately supports APOSCREEN** |  |  |  |  |  |  |  |  |  |

**For each statement please select an answer that best suits your experience using Option A. If the statement is not relevant to you please select an answer from Option B.**

|  | | **Option A** | | | | |  | **Option B** | | |
| --- | --- | --- | --- | --- | --- | --- | --- | --- | --- | --- |
| **Section C4** | | **Strongly Agree** | **Agree** | **Neither agree nor disagree** | **Disagree** | **Strongly disagree** |  | **Not relevant to my role** | **Not relevant at this stage** | **Not relevant to the intervention** |
| **1.** | **I am aware of reports about the effects of APOSCREEN** |  |  |  |  |  |  |  |  |  |
| **2.** | **The staff agree that APOSCREEN is worthwhile** |  |  |  |  |  |  |  |  |  |
| **3.** | **I value the effects that APOSCREEN has had on my work** |  |  |  |  |  |  |  |  |  |
| **4.** | **Feedback about APOSCREEN can be used to improve it in the future** |  |  |  |  |  |  |  |  |  |
| **5.** | **I can modify how I work with APOSCREEN** |  |  |  |  |  |  |  |  |  |
