# Supplementary material for: APOSCREEN-1 – a prospective, single-arm clinical trial for the implementation of a pharmacy-based screening for cardiovascular-kidney-metabolic risk factors in Schleswig-Holstein
Source: BMC Nephrol. 2026 Jun 5;27:357. doi: 10.1186/s12882-026-05090-x (PMC13244955; doi:10.1186/s12882-026-05090-x)
Supplement: Supplementary file 6 — Supplementary Material 6 [file 12882_2026_5090_MOESM6_ESM.docx]

**TFA Questionnaire**

| Item | TFA questionnaire items |
| --- | --- |
| **Affective attitude** | Did you like or dislike the APOSCREEN-1 screening?   \| Strongly dislike \| Dislike \| No opinion \| Like \| Strongly like \| \| --- \| --- \| --- \| --- \| --- \| \| 1 \| **2** \| **3** \| **4** \| **5** \| |
| **Burden** | How much effort did it take complete the APOSCREEN-1 screening?   \| No effort at all \| A little effort \| No opinion \| A lot of effort \| Huge effort \| \| --- \| --- \| --- \| --- \| --- \| \| 1 \| **2** \| **3** \| **4** \| **5** \| |
| **Ethicality** | There are moral or ethical consequences to undergo the APOSCREEN-1 screening.   \| Strongly disagree \| Disagree \| No opinion \| Agree \| Strongly agree \| \| --- \| --- \| --- \| --- \| --- \| \| 1 \| **2** \| **3** \| **4** \| **5** \| |
| **Perceived effectiveness** | The APOSCREEN-1 screening has improved my chances of not having an undetected risk factor for cardiovascular, kidney, or metabolic risk factors.   \| Strongly disagree \| Disagree \| No opinion \| Agree \| Strongly agree \| \| --- \| --- \| --- \| --- \| --- \| \| 1 \| **2** \| **3** \| **4** \| **5** \| |
| **Intervention coherence** | It is clear to me how the APOSCREEN-1 screening will help to reduce my risk of having an undetected risk factor for cardiovascular, kidney, or metabolic diseases.   \| Strongly disagree \| Disagree \| No opinion \| Agree \| Strongly agree \| \| --- \| --- \| --- \| --- \| --- \| \| 1 \| **2** \| **3** \| **4** \| **5** \| |
| **Self -efficacy** | How confident did you feel about completing the APOSCREEN-1 screening?   \| Very unconfident \| Unconfident \| No opinion \| Confident \| Very confident \| \| --- \| --- \| --- \| --- \| --- \| \| 1 \| **2** \| **3** \| **4** \| **5** \| |
| **Opportunity costs** | The APOSCREEN-1 screening interfered with my other priorities   \| Strongly disagree \| Disagree \| No opinion \| Agree \| Strongly agree \| \| --- \| --- \| --- \| --- \| --- \| \| 1 \| **2** \| **3** \| **4** \| **5** \| |
| **General acceptability** | How acceptable was the APOSCREEN-1 screening to you?   \| Completely unacceptable \| Unacceptable \| No opinion \| Acceptable \| Completely acceptable \| \| --- \| --- \| --- \| --- \| --- \| \| 1 \| **2** \| **3** \| **4** \| **5** \| |
